# Announcing the *Nanoscale Advances* Paper Prize

**DOI:** 10.1039/d5na90081f

**Published:** 2025-12-12

**Authors:** Paul Scott, Jeremy P. Allen, Yue Zhang, Dirk M. Guldi

**Affiliations:** a Royal Society of Chemistry Thomas Graham House, Science Park, Milton Road Cambridge UK; b School of Materials Science and Engineering, Beijing Key Laboratory for Advanced Energy Materials and Technologies, University of Science and Technology Beijing Beijing 100083 People's Republic of China; c Academy for Advanced Interdisciplinary Science and Technology, Key Laboratory of Advanced Materials and Devices for Post-Moore Chips Ministry of Education, State Key Laboratory for Advanced Metals and Materials, University of Science and Technology Beijing Beijing 100083 People's Republic of China; d Department of Chemistry and Pharmacy, Interdisciplinary Center for Molecular Materials (ICMM), Physical Chemistry I, Friedrich-Alexander-Universität Erlangen-Nürnberg Erlangen Germany

## Abstract

Here at *Nanoscale Advances* we are lucky to receive high quality research papers from across the full range of nanoscience and nanotechnology topics every year. We wanted to find a way to recognise the most significant papers published in the journal each year, judged by the expert nanoscience and nanotechnology researchers who make up our Editorial and Advisory Boards. In this article we are excited to announce the winner and runners-up of the very first Paper Prize, as well as the process that we have taken to select these excellent articles.

## Abstract 2

We are delighted to announce the winners of the inaugural *Nanoscale Advances* Paper Prize.

## Graphical abstract

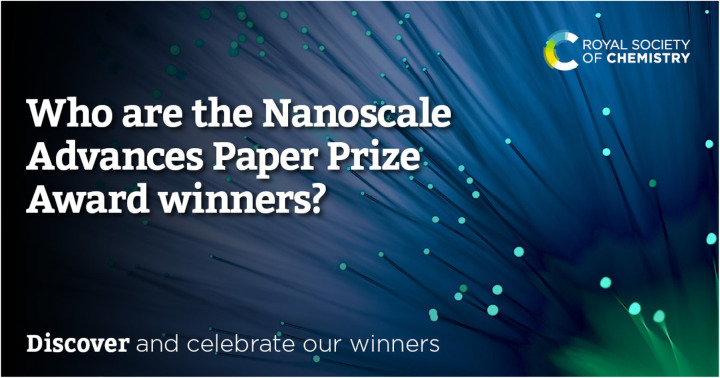

## 2025 *Nanoscale Advances* Paper Prize winners

### Winning paper

Enhancing photoluminescence performance of perovskite quantum dots with plasmonic nanoparticles: insights into mechanisms and light-emitting applications (https://doi.org/10.1039/D3NA01078C) by Gautham Kumar, Chien-Chung Lin, Hao-Chung Kuo and Fang-Chung Chen

### Runner-up papers

Understanding the effects of ethanol on the liposome bilayer structure using microfluidic-based time-resolved small-angle X-ray scattering and molecular dynamics simulations (https://doi.org/10.1039/D3NA01073B) by Masatoshi Maeki, Niko Kimura, Yuto Okada, Kazuki Shimizu, Kana Shibata, Yusuke Miyazaki, Akihiko Ishida, Kento Yonezawa, Nobutaka Shimizu, Wataru Shinoda and Manabu Tokeshi

Helical interfacial modulation for perovskite photovoltaics (https://doi.org/10.1039/D4NA00027G) by Ghewa AlSabeh, Masaud Almalki, Sitthichok Kasemthaveechok, Marco A. Ruiz-Preciado, Hong Zhang, Nicolas Vanthuyne, Paul Zimmermann, Daphne M. Dekker, Felix Thomas Eickemeyer, Alexander Hinderhofer, Frank Schreiber, Shaik M. Zakeeruddin, Bruno Ehrler, Jeanne Crassous, Jovana V. Milić and Michael Grätzel

Recipients of the Paper Prize will receive:

• The opportunity to present during a Royal Society of Chemistry-run webinar

• Branded mugs for each author involved in the work

• Certificates for all authors who contributed to the work

• Promotion through the various journal channels.

### Selection process

We want this award to be transparent and inclusive, representing our nanoscience and nanotechnology community. With that in mind, please don't hesitate to get in touch if you have any suggestions for how we can improve the process in future years (nanoscaleadvances-rsc@rsc.org).

A shortlist of papers was selected by the Editorial Office using quantitative measures taking a snapshot of the performance of all papers published in a 2024 issue. Citations, article downloads, and Altmetric scores (social media and news engagement) were considered. This data was gathered in April 2025, and the length of time since online publication was taken into account during our assessment to avoid the selection automatically favouring those papers that were published first. We also asked our Editorial Board members to recommend papers to us that they recalled seeing, either as a reader or as a handling editor.

The shortlist of 12 papers was then sent to our Editorial Board and Advisory Board for voting. This diverse group are all expert nanoscience and nanotechnology researchers and between them cover the wide-ranging scope of the journal. We felt that this maximised the subject area expertise of those who were voting and was the fairest method of selecting a single winning paper.

They were asked to judge the papers based on the following criteria:

• Quality of discovery/advance presented

• Potential future impact

• Originality of the work

• Significance to the field

Selecting a single paper to recognise for this annual award necessarily involved a degree of subjective assessment based on the above criteria. However, the broad experience and knowledge of our combined Editorial and Advisory Board members allowed a clear consensus to be reached. Board members were asked to indicate any potential conflicts of interest and these were taken into account.

### Inclusion and diversity considerations

The Royal Society of Chemistry is committed to supporting and improving inclusion and diversity in the chemical sciences, and this extends to our paper prizes. Our intention with the Paper Prize over time is to ensure that it is representative of the community as a whole. We recognise that we need to do more to foster inclusion and diversity in all areas of the publishing process. Along with over 30 other publishing organisations, we have made a joint commitment for action to set a new standard to ensure a more inclusive and diverse culture within scholarly publishing. The resources we provide on our webpage (https://www.rsc.org/new-perspectives/talent/inclusion-and-diversity/resources/) are available for all to use to explain how we define equality, diversity and inclusion as well as implicit bias.

### Future plans

We plan to award a *Nanoscale Advances* Paper Prize on an annual basis going forward. To be in with a chance of winning please consider publishing your next nanoscience or nanotechnology work in the journal.

